# Hormonal Control of Blood Viscosity

**DOI:** 10.7759/cureus.55237

**Published:** 2024-02-29

**Authors:** Gregory D Sloop, Gheorghe Pop, Joseph J Weidman, John A St. Cyr

**Affiliations:** 1 Pathology, Idaho College of Osteopathic Medicine, Meridian, USA; 2 Cardiology, Radboud University Medical Center, Nijmegen, NLD; 3 Internal Medicine, Independent Researcher, Columbia, USA; 4 Cardiac/Thoracic/Vascular Surgery, Jacqmar, Inc., Minneapolis, USA

**Keywords:** renin-angiotensin-aldosterone system, prostaglandin e2, nitric oxide, erythrocyte, hemodynamics, erythrocyte aggregation, erythrocyte deformability, systemic vascular resistance response, carotid sinus, blood viscosity

## Abstract

The hemodynamic milieu differs throughout the vascular tree because of varying vascular geometry and blood velocities. Accordingly, the risk of turbulence, which is dictated by the Reynolds and Dean numbers, also varies. Relatively high blood viscosity is needed to prevent turbulence in the left ventricle and aorta, where high-velocity blood changes direction several times. Low blood viscosity is needed in the capillaries, where erythrocytes pass through vessels with a diameter smaller than their own. In addition, higher blood viscosity is necessary when the cardiac output and peak blood velocity increase as a part of a sympathetic response or anemia, which occurs following significant hemorrhage. Blood viscosity, as reflected in systemic vascular resistance and vascular wall shear stress, is sensed, respectively, by cardiomyocyte stretching in the left ventricle and mechanoreceptors for wall shear stress in the carotid sinus. By controlling blood volume and red blood cell mass, the renin-aldosterone-angiotensin system and the systemic vascular resistance response control the hematocrit, the strongest intrinsic determinant of blood viscosity. These responses provide gross control of blood viscosity. Fine-tuning of blood viscosity in transient conditions is provided by hormonal control of erythrocyte deformability. The short half-life of some of these hormones limits their activity to specific vascular beds. Hormones that modulate blood viscosity include erythropoietin, angiotensin II, brain natriuretic factor, epinephrine, prostacyclin E2, antidiuretic hormone, and nitric oxide.

## Introduction and background

Blood viscosity is determined by both extrinsic and intrinsic factors. Extrinsic factors include the shear rate and body temperature. The shear rate correlates roughly with blood velocity: when the flow is rapid, the shear rate is high, and it is low when the flow is sluggish. The dependence of blood viscosity on the shear rate is due to reversible erythrocyte deformation and aggregation, which makes blood a “non-Newtonian fluid” [1]. In general, increased temperature decreases the viscosity of fluids. In humans, the decrease in viscosity with increased temperature [2] is useful to reduce the increased blood viscosity caused by inflammation [3].

The intrinsic factors that determine blood viscosity are the hematocrit, erythrocyte deformability, and the plasma concentrations of certain components, especially fibrinogen, low-density lipoprotein (LDL), high-density lipoprotein (HDL), and albumin. Large proteins and lipoprotein particles can act like glue and, by simultaneously binding to two erythrocytes, create erythrocyte aggregates. HDL is too small to simultaneously bind two erythrocytes; instead, it antagonizes erythrocyte aggregation and lowers blood viscosity by competing with LDL for binding sites on the erythrocyte [4]. The forces binding erythrocytes are weak and aggregates are readily dispersed with increasing shear. Thus, erythrocyte aggregations have the greatest effect on blood viscosity at low shear rates. Erythrocyte deformability has the greatest effect on blood viscosity at high shear rates. Shear rate and hematocrit are the strongest factors in determining blood viscosity.

## Review

Why is blood viscosity important?

Blood viscosity is a part of what the eminent physiologist Claude Bernard called the “milieu intérieur,” i.e., the condition created by fundamental parameters, such as body temperature, pH, and plasma osmolarity [1]. These parameters must be maintained within narrow limits to preserve health. There are several reasons why the viscosity of blood is so important. First, a change in blood viscosity causes a threefold inverse change in blood flow [5]. Thus, blood viscosity influences organ perfusion, which is an important determinant of organ function. An example is the increase in the plasma concentration of creatinine when blood viscosity increases due to paraproteinemia [6].

Similarly, glucose intolerance can be caused by elevated blood viscosity. By reducing blood flow and perfusion of skeletal muscle, elevated blood viscosity reduces postprandial uptake of glucose by skeletal muscle. Skeletal muscle normally takes up approximately 80% of circulating glucose following a meal. Thus, elevated blood viscosity and reduced perfusion of skeletal muscle leads to glucose intolerance, metabolic syndrome, and diabetes mellitus type 2 [7]. In addition, elevated blood viscosity plays an etiologic role in atherothrombosis and leads to both arterial and venous thrombosis. According to the thrombogenic theory of atherothrombosis, atherosclerotic plaques are organized arterial mural thrombi [8].

Normal blood viscosity is necessary for the preferential flow patterns within the heart [1]. These are the stereotypical flow streamlines that occur when cardiac anatomy and blood viscosity are normal. They minimize the crossing of flow streamlines, which reduces friction, thereby allowing blood flow with the least expenditure of energy. Elevated blood viscosity, which occurs with inflammation that accompanies COVID-19, results in the loss of preferential flow patterns and intraventricular thrombosis despite normal wall motion [9].

Normal blood viscosity is necessary to maintain the antithrombotic phenotype of the endothelium. Normal blood viscosity and wall shear stress cause endothelial cells to express nitric oxide and prostacyclin, molecules with antiplatelet activity. Rudolph Virchow, the father of pathology, first identified sluggish blood flow as a cause of thrombosis. Sluggish blood flow is a manifestation of increased blood viscosity [3].

High-velocity blood delivers oxygen and metabolic substrates faster than low-velocity blood. However, the risk of turbulence increases with higher blood velocities. Turbulence causes hemolysis, activates platelets and neutrophils, and increases the dissipation of energy. Uncontrolled hemolysis can cause a life-threatening vicious cycle: hemolysis lowers blood viscosity, which increases blood velocity and further worsens turbulence and hemolysis until fatal high-output cardiac failure and anemia occur [10]. This imposes a lower limit to normal blood viscosity.

In a straight tube, the propensity for the transition from laminar to turbulent flow is described by the Reynolds number (Re), which is directly related to blood velocity and inversely related to blood viscosity [11]. The larger the value of Re, the greater the chance of losing laminar flow. Therefore, adequate blood viscosity will prevent turbulence.

In areas of changing vascular geometry, such as branches and curves, the requirements for laminar flow are much more stringent because the momentum of erythrocytes causes them to resist changes in direction. Momentum is the product of velocity and mass. The propensity for the transition from laminar to turbulent flow in curved tubes is described by the Dean number (De), which incorporates the Reynolds number and is also inversely related to viscosity [11]. Turbulence develops at much lower velocities than in straight tubes, necessitating higher blood viscosity.

The hemodynamic milieu varies throughout the vascular tree. An erythrocyte takes approximately one minute to complete a circuit through the circulation. During its journey, it encounters a variety of hemodynamic milieus. Thus, a single viscosity of blood is not optimal for the entire vascular tree. A high-velocity flow is present in the left ventricular outflow tract. Immediately after leaving the heart, the erythrocyte encounters the curve of the aortic arch and several large acute branches, the brachiocephalic, left common carotid, and left subclavian arteries. During diastole, blood flows through the coronary arteries, over the convexity of the heart, and encounters the acute-angled branches of the epicardial coronary arteries. A higher blood viscosity is appropriate because of the changing arterial geometry in these circulations.

Friction between erythrocytes and endothelial cells slows blood flow. When erythrocytes reach the capillaries, they must deform to traverse vessels with a diameter smaller than their own. Low viscosity is optimal when resistance is high as in capillaries. On the venous side, blood velocity increases because of skeletal muscle contraction but does not reach arterial values. High-velocity blood is present in the pulmonary arteries.

The intervillous space of the placenta is a distinct hemodynamic milieu with low shear conditions. In pre-eclampsia, blood viscosity is increased especially at low shear rates because of increased erythrocyte aggregation. This compromises fetal perfusion. A study of maternal plasma volume expansion in pre-eclampsia using pasteurized plasma reduced maternal blood viscosity, blood pressure, and peripheral vascular resistance [12]. 

Control of blood viscosity

As befitting an important parameter, blood viscosity is tightly controlled. Gross control of blood viscosity is accomplished by modulation of the hematocrit, the strongest intrinsic determinant of blood viscosity. The hematocrit is modulated by altering plasma volume and red blood cell mass. Many physiologic functions are controlled by antagonistic pairs. Insulin and glucagon and the sympathetic and parasympathetic nervous systems come immediately to mind. The renin-angiotensin-aldosterone system (RAAS) and the systemic vascular resistance response (SVRR) are another antagonistic pair [13]. The former causes vasoconstriction and increases plasma volume and red cell mass while the latter does the opposite. Brain natriuretic peptide (BNP), secreted by left ventricular myocytes as a part of the SVRR, antagonizes the sensation of thirst caused by angiotensin II, an effector of the RAAS [14].

The activity of the RAAS in causing sodium retention and increasing plasma volume is well known. Its activity in increasing red blood cell mass has received less attention. Angiotensin II is a secretagogue of erythropoietin and a growth factor for erythroid progenitors. The most dramatic example of the effect of the RAAS on erythropoiesis is posttransplant erythrocytosis [15]. This is defined as a hematocrit >51% or a hemoglobin concentration >17 mg/dL after renal transplantation. Morbidity attributable to hyperviscosity is common. Approximately 60% of patients experience malaise, headache, lethargy, and dizziness. Ten to 30% of patients develop thrombotic events. Blockade of the RAAS is the most effective therapy.

The activity of the RAAS is modulated by the output from the carotid sinus. This is a dilation of the proximal internal carotid artery. This dilation causes an area of recirculation to develop, which magnifies wall shear stress. Wall shear stress is detected by endothelial mechanoreceptors. Neural output from the carotid sinus is transmitted to the brainstem via the glossopharyngeal nerve (cranial nerve IX) [16], where it influences sympathetic output to the kidney.

The SVRR was described in 2015 [13]. In this response, systemic vascular resistance is detected by stretch receptors in the left ventricle. This causes the release of brain natriuretic factor from cardiomyocytes, resulting in vasodilation and natriuresis. It also decreases red blood cell mass via several mechanisms. A soluble erythropoietin receptor is produced, which binds and eliminates circulating erythropoietin before the hormone can bind to erythroid precursors in the bone marrow. The soluble erythropoietin receptor is a truncated form of the normal erythropoietin receptors, which lacks the transmembrane and intracellular domains. Expression of only the soluble portion of a receptor to serve as a “decoy receptor” is a common strategy in class I cytokine receptors. The soluble erythropoietin receptor immediately decreases concentrations of circulating erythropoietin. This activates splenic macrophages, which phagocytize erythrocytes.

The soluble erythropoietin receptor causes erythropoietin resistance, the phenomenon in which increased doses of exogenous erythropoietin are necessary to maintain a target hematocrit in renal failure patients. Erythropoietin resistance is a manifestation of the SVRR to prevent complications of hyperviscosity, such as hypertension and thrombosis. In addition, the SVRR causes decreased transcription of the erythropoietin gene.

The SVRR can cause clinical anemia [6,13]. Examples are the anemias seen in multiple myeloma and other myeloproliferative disorders, lymphoproliferative disorders, some acute and chronic infections, and renal failure. Elevated blood viscosity would develop in these conditions if not for the superimposed anemia. The SVRR also causes the anemias of low-output heart failure and spaceflight, which are discussed below.

In addition, blood viscosity is controlled by hormonal modulation of erythrocyte deformability. The half-life of a hormone determines whether it affects erythrocyte deformability and blood viscosity throughout the circulation or only a limited area. Epinephrine decreases erythrocyte deformability [17] and has a half-life greater than one minute. Thus, it increases blood viscosity throughout the circulation, which is appropriate when blood velocity increases because of the elevated heart rate and inotropic state caused by a sympathetic response.

Regional control of blood viscosity is accomplished via hormones with shorter half-lives. Higher viscosity is necessary to prevent turbulence when blood velocity is high and the direction of flow changes, as in the left ventricle and proximal aorta. PGE2 is secreted by alveolar type 2 pneumocytes and diffuses into alveolar capillaries [6]. It decreases erythrocyte deformability in vitro [17]. Its activity in the bloodstream is limited by its short half-life, 10 to 30 seconds. Thus, PGE2 increases blood viscosity in the pulmonary vein, the left side of the heart, and the aortic arch, where high-velocity flow constantly changes direction. This minimizes turbulence, although a small amount of energy is normally lost to turbulence in healthy individuals [18]. Energy loss increases with higher blood velocity as seen with anemia, exercise, and “the fight or flight” response.

Nitric oxide is produced by both endothelial cells and erythrocytes. Shear stress in the physiologic range induces endothelial production of nitric oxide. This dilates the microvasculature. Erythrocyte-produced nitric oxide, also driven by shear stress, also appears to increase erythrocyte deformability [19]. Intravascular nitric oxide has an extremely short half-life, roughly 2 milliseconds. Hormones that affect blood viscosity are listed in Table 1.

**Table 1:** Hormonal control of blood viscosity RAAS: renin-angiotensin-aldosterone system; RBC: red blood cell; SVRR: systemic vascular resistance response; PGE2: prostaglandin E2; NO: nitric oxide

Axis/hormone	Effect on blood viscosity	Mechanism
RAAS	Increase	Increase RBC mass
SVRR	Decrease	Decrease RBC mass
Epinephrine	Increase	Decrease RBC deformability
Vasopressin	Decrease	Increase plasma volume
PGE2	Increase	Decrease RBC deformability
NO	Decrease	Increase erythrocyte deformability

Low-output cardiac failure

When cardiac power decreases as in low-output heart failure, decreased renal blood flow results in decreased removal of free water, increasing left ventricular end-diastolic volume. This causes stretch receptors in the left ventricle to fire and increases the activity of the SVRR. This reduces the hematocrit, systemic vascular resistance, and the burden on the failing left ventricle [20,13]. This establishes a new state of homeostasis. If cardiac power improves, wall shear stress in the carotid sinus will increase and upregulate the activity of the RAAS.

Anemia of spaceflight

The activity of both the SVRR and RAAS are seen in space flight anemia. Release from Earth’s gravity causes a shift of fluid from gravity-dependent areas into the central circulation. This increases intravascular volume and activates the SVRR, reducing red blood cell mass. If the duration of space flight is short, repletion of intravascular volume by the RAAS following the return to Earth occurs more quickly than the restoration of red blood mass, resulting in anemia.

Hemodynamically significant blood loss

Hemodynamically significant blood loss decreases intravascular hydrostatic pressure. The lost intravascular volume is quickly restored due to passive flow from the extravascular space. This causes dilutional anemia, which reduces blood viscosity. Reduced wall shear stress is detected in the carotid sinus. Information from the carotid sinus is relayed to the central nervous system and causes sympathetic tone to increase [21], which increases cardiac output to maintain oxygen transport. Epinephrine decreases erythrocyte deformability [17] and increases cardiac contractility, increasing blood viscosity and peak blood velocity. These increase wall shear stress in the carotid sinus, activating the RAAS. Aldosterone causes sodium retention and angiotensin II increases red cell mass. As a result, the hematocrit and total body water are restored.

Sports anemia

Sports anemia is a dilutional anemia present in some extremely well-conditioned athletes. It is a dilutional anemia, in which plasma volume is increased disproportionately to red blood cell mass. Improving fitness decreases systemic vascular resistance by 7.1%, increases stroke volume by 15.4%, and increases left ventricular mass by 17% [22,23]. These changes result in increased cardiac power, peak arterial blood velocity, and wall shear rate in the carotid sinus.

This is detected by mechanoreceptors in the carotid sinus, allowing a physiologic response to maintain homeostasis. This allows a complex response in which plasma volume is increased disproportionately to red blood cell mass. This decreases blood viscosity and normalizes arterial wall shear stress. Sports anemia should be viewed as an adaptive response, not a pathological condition. Dilutional anemia is also seen in successful pregnancies.

Diurnal variation in blood volume

Glomerular filtration and urine production continue during sleep. Because there is no oral fluid intake during this period, hemoconcentration ensues. Moreover, posture results in a fluid shift into the interstitial space, worsening hemoconcentration. This causes circadian variation in certain hematological variables. Blood viscosity and hematocrit follow similar circadian patterns. In one study, diurnal variation of blood viscosity increased with decreasing shear rates once the daily routine was established. At the low shear rate of 2.3-1, blood viscosity varied between 62% and 136% from the mean value over 24 hours. Blood viscosity and hematocrit peaked at 0800 hours, which may help explain the increase in acute myocardial events and strokes noted in the morning hours [24,25]. Plasma vasopressin concentrations are maximal at night to minimize the loss of free water and hemoconcentration during sleep [26]. The output from the carotid sinus as a part of the RAAS upregulates thirst [14] to restore intravascular volume.

How should blood viscosity be measured?

Blood is a suspension in which many of the suspended particles have the remarkable ability to change their deformability and thus the viscosity of the suspension. This happens continuously throughout the circulation in response to hemorheological conditions. A man-made comparison does not immediately come to mind. How should one measure the viscosity of a remarkable fluid? Whole blood is by far the most complex specimen encountered in the clinical laboratory. Plasma, serum, cerebrospinal fluid, and synovial fluid are much less complicated.

The optimal specimen is peripheral venous blood for two reasons: it is easily accessible, and a large body of work has already been performed on this specimen. A normal range for males and females has been established [11]. As blood viscosity is a non-Newtonian fluid, its viscosity varies with shear rate. Therefore, when the viscosity of whole blood is described, the shear rate it has been measured should always be mentioned. The existence of a normal range allows the relative risk in a patient for viscosity-associated conditions, such as atherosclerotic cardiovascular disease, deep venous thrombosis, and sudden deafness to be estimated. Analysis of venous blood has revealed that elevated blood viscosity is associated with most of the major risk factors for atherosclerotic cardiovascular disease, including male sex, hyperlipidemia, hypertension, diabetes mellitus, and cigarette smoking. Because an increase in blood viscosity causes a threefold inverse change to blood flow, increased blood viscosity predisposes to sluggish blood flow and thrombosis as Virchow noted in the 19th century [3,8]. The thrombogenic theory of atherogenesis holds that atherosclerotic plaques are organized mural thrombi. It has been suggested that increased blood viscosity is a common pathway by which most common risk factors accelerate atherosclerotic cardiovascular disease [8].

Furthermore, studies on peripheral venous blood revealed that acute inflammation is associated with increased blood viscosity. The reason is that during inflammation the concentration of acute-phase proteins rises, these proteins, especially fibrinogen, act as a glue between erythrocytes, which enhances aggregation and subsequently blood viscosity. This explains the increased risk of deep venous thrombosis and myocardial infarction complicating a wide variety of infectious diseases and even immunizations [3].

Studies on peripheral venous blood also revealed that the anemia accompanying falciparum malaria is actually a homeostatic response to normalize blood viscosity. Multiple lines of evidence suggest that many other hemolytic anemias, such as sickle cell anemia and hereditary spherocytosis, are also compensatory to normalize blood viscosity [13]. As indicated above, the anemia accompanying low-output cardiac failure is also compensatory [20].

This review demonstrates that erythrocyte deformability and whole-blood viscosity are inextricably linked. A failure of the erythrocyte to assume the proper degree of deformability could lead to hyperviscosity and thus hypertension [27]. Several reports have documented decreased erythrocyte deformability in association with hypertension (reviewed in reference [28]). Genome-wide association studies have identified more than 1477 single-nucleotide polymorphisms (SNPs) associated with blood pressure traits [29]. The pathologic effect of the majority of these SNPs is unknown. Given that a new erythrocyte transmembrane channel was described as recently as 2021, a recent review of erythrocyte transmembrane channels argues that “We are only at the beginning to [sic] understand the role of ion channels as molecular regulators or modulators of RBC channelopathies" [30]. Unfortunately, erythrocyte deformability was not one of the traits measured in the aforementioned genome-wide association study. Such a study should be undertaken to uncover any abnormalities in erythrocyte deformability in primary hypertension.

## Conclusions

Gross control of blood viscosity is accomplished by the modulation of the erythrocyte mass and plasma volume. This is accomplished by the RAAS and SVRR. Information to control these hormonal axes is collected by endothelial mechanoreceptors in the carotid sinus and stretch mechanoreceptors in the left ventricle, respectively. The hemodynamic milieu varies with a sympathetic tone and the anatomic region. In response, hormonal modulation of erythrocyte deformability allows homeostasis of endothelial wall shear stress.
